# Lifelong learning practices and leisure-time exercise habits of academic and community-based physicians

**DOI:** 10.15694/mep.2019.000021.1

**Published:** 2019-01-29

**Authors:** Oksana Babenko, Mao Ding, Sudha Koppula

**Affiliations:** 1 University of Alberta

**Keywords:** lifelong learning, leisure-time exercise, physician wellbeing

## Abstract

This article was migrated. The article was marked as recommended.

**Objective**: Physicians are required to be lifelong learners for the provision of quality patient care. At the same time, physician wellbeing is a critical component in the delivery of such care. This study was designed to examine: (1) lifelong learning practices and leisure-time exercise habits of academic and community-based physicians; and (2) associations of leisure-time exercise with work engagement, exhaustion, and professional life satisfaction.

**Methods**: Using an online survey, quantitative data were collected from physicians practicing in Canada. The survey contained validated scales of physician lifelong learning, leisure-time exercise, work engagement, work exhaustion, and professional life satisfaction. Descriptive, chi-square, t-test, and correlational analyses were performed.

**Results**: Physicians (n=57) reported moderately high levels of lifelong learning, with no significant difference between academic and community-based physicians. To stay current in their practice, the majority of physicians reported exchanging ideas/asking colleagues and searching databases as questions arise (>90%), followed by engaging in clinical teaching and attending conferences and meetings of professional organizations (>80%). Watching podcasts and webinars was the least preferred lifelong learning activity (<50%). With respect to leisure-time exercise habits, more community-based physicians reported engaging in mild and/or moderate forms of exercising, whereas more academic physicians reported engaging in strenuous exercising in a typical week. Correlational analyses revealed that physicians’ leisure-time exercise scores were positively correlated with professional life satisfaction (r = 0.25; p = 0.058) and work engagement (r = 0.29; p = 0.028) and negatively correlated with work exhaustion (r = −0.34; p = 0.01).

**Conclusions**: Irrespective of the practice type, physicians tend to engage in lifelong learning activities that offer in-person interactions with colleagues and trainees. Regular participation in leisure-time exercise appears to enhance physicians’ professional wellbeing. As such, these activities and habits should be encouraged, supported, and promoted within institutional culture and health systems in general.

## Introduction

In order to provide quality patient care, physicians need to maintain currency with new evidence, technological advances, and guidelines that are continuously being developed and updated. To this end, professional organizations have identified lifelong learning as a core competency in health professions and require practitioners to be engaged in continuing professional development (CPD) ( American Association of Colleges of Nursing and Association of American Medical Colleges, 2010; Interprofessional Education Collaborative Expert Panel, 2011; Frank, Snell and Sherbino, 2015). Hojat and colleagues defined lifelong learning as “an attribute involving a set of self-initiated activities and information-seeking skills with sustained motivation to learn and the ability to recognize one’s own learning needs.” ( Hojat, Veloski and Gonnella, 2009, p. 1066) Lifelong learning is a recognized indicator of physician professionalism ( Hojat, Veloski and Gonnella, 2009; American Association of Colleges of Nursing and Association of American Medical Colleges, 2010; Interprofessional Education Collaborative Expert Panel, 2011; Frank, Snell and Sherbino, 2015) and needs to be cultivated as early as in medical training ( Babenko *et al*., 2017).

At the same time, physician wellbeing is a critical, but often overlooked, component in the provision of quality patient care. Despite the well-recognized health benefits of physical activity and the importance of leisure for quality of life, research has shown that physicians’ exercise habits start to deteriorate upon commencing medical training and decline over the course of their careers ( Gnanendran *et al*., 2011; Klein, Guenther and Ross, 2016). This finding is alarming because physicians’ participation in physical activity and leisure not only benefit their own health but also makes their endorsement of an active lifestyle more credible and patient counseling more effective ( Gnanendran *et al*., 2011; Klein, Guenther and Ross, 2016). In a sense, physician wellbeing is an indicator of professionalism. However, while professional organizations do encourage physicians to engage in physical activity and leisure-time exercise, these are not formal professional requirements.

All in all, when health care providers do not keep up with their changing context and/or experience poor wellbeing, it impacts the quality of clinical care and patient safety ( Dyrbye *et al*., 2013). This study was designed to examine lifelong learning practices and leisure-time exercise habits of academic and community-based physicians. We also aimed to examine associations of physicians’ leisure-time exercise with work engagement, exhaustion, and professional life satisfaction, all of which are important for physician wellbeing and, by extension, for the provision of quality patient care.

## Methods

### Study design, procedure, and participants

This study is part of a larger research project investigating personal and contextual factors in learning and wellbeing of medical students and practicing physicians. The link to the online questionnaire was circulated using professional mailing lists and word of mouth, including announcements at professional events (e.g., conferences). Participation in the study was voluntary and participants had the option not to respond to a question if they did not feel comfortable. Informed consent was implied by the overt action of completing the electronic questionnaire after reading the information letter. Ethics approval was obtained from the Institutional Research Ethics Board (Pro00066510). Fifty-seven physicians practicing in Canada completed the survey. Each physician was asked if she/he considered her-/himself an academic practitioner or a community-based practitioner. Eleven participants, however, chose not to indicate their practice type. Of those who indicated their practice type, 27 (58.7%) physicians identified themselves as community-based practitioners and 19 (41.3%) as academic practitioners.

### Measures

*Lifelong learning:* The 14-item Jefferson Scale of Physician Lifelong Learning (JeffSPLL; Hojat, Veloski and Gonnella, 2009) was used to measure physician lifelong learning, including learning beliefs, attention to learning opportunities, self-initiated activities, and information-seeking skills. Sample items are: “I believe I would fall behind if I stopped learning about new developments in my profession” and “I regularly make time for self-directed learning, even when I have a busy practice schedule and other professional and family obligations”. Participants responded to items using a four-point Likert-type scale (1-strongly disagree; 4-strongly agree). Higher scale scores were indicative of greater lifelong learning. Physicians were also asked to indicate what they do to update their knowledge and skills and to stay current in their practice by selecting from a list of activities.

*Leisure-time exercise:* The Godin Leisure-Time Exercise Questionnaire (GLTE; Godin and Shephard, 1997) was used to measure physicians’ leisure-time exercise habits. Using one of the response options (never; 1-3 times a week; 4-6 times a week; 7 times a week or more), physicians were asked to indicate the number of times they engaged in mild, moderate, and strenuous leisure-time exercise bouts of at least 15 min duration in a typical week; examples of such activities were provided for each intensity category. The number of bouts at each intensity was then multiplied by 3, 5, and 9 metabolic equivalents (for mild, moderate, and strenuous activity, respectively) and summed to derive a leisure-time exercise score for each physician.

*Work engagement and exhaustion:* The 16-item Oldenburg Burnout Inventory (OLBI; Demerouti and Bakker, 2008; Demerouti, Mostert and Bakker, 2010), which consists of two scales, was used to measure work engagement and exhaustion. Using a four-point Likert-type scale (1-strongly disagree; 4-strongly agree), physicians were asked to indicate the level of agreement with each statement in relation to their work. Sample items are: “I find my work to be a positive challenge” (engagement) and “After work, I tend to need more time than in the past to relax and feel better” (exhaustion). Higher scores on each scale were indicative of greater work engagement and exhaustion, respectively.

*Professional life satisfaction:* The five-item Satisfaction with Life Scale ( SWLS; Diener *et al*.,1985) was used to assess overall satisfaction with professional life. A minor modification was employed for this study, with the word “professional” added in each item (i.e., professional life) to prompt responding to professional life, rather than life more globally. Using a seven-point Likert-type scale (1-strongly disagree; 7-strongly agree), physicians were asked to indicate the degree of agreement with each statement in relation to their professional life. Sample items are: “In most ways, my professional life is close to my ideal” and “If I could restart my professional life, I would change almost nothing”. Higher scale scores were indicative of greater satisfaction with professional life.

### Analyses

SPSS 25.0 was used to analyze the data. Descriptive statistics (frequencies, means, standard deviations) were computed for all study variables. The chi-square test was used in the analyses of categorical variables; the independent samples t-test was used in the analyses of continuous variables, specifically to examine differences in physician lifelong learning and leisure-time exercise scores based on the practice type (i.e., academic vs. community-based). Correlational analyses were performed to examine associations of physicians’ leisure-time exercise with work engagement, work exhaustion, and professional life satisfaction.

## Results/Analysis

Results of the analyses of physician lifelong learning are shown in Table 1. On average, physicians reported moderately high levels of lifelong learning. No significant difference was observed in the lifelong learning scores of academic and community-based physicians. With respect to specific lifelong learning activities, the majority of academic and community-based physicians reported exchanging ideas/asking colleagues and searching databases as questions arise (>90%), followed by engaging in clinical teaching and attending conferences and meetings of professional organizations (>80%). Watching podcasts and webinars was the least preferred lifelong learning activity for both academic and community-based physicians (<50%). A greater number of academic physicians than community-based physicians reported engaging in research/scholarly activities.

**Table 1.** Physician lifelong learning by practice type

**Variables**	Academic, n=19	Community-based, n=27	p-value
Lifelong learning, M (SD)	3.29 (0.27)	3.34 (0.40)	0.63
Lifelong learning activities, n (%)			
- Exchange ideas/ask my colleagues	19 (100)	27 (100)	na
- Search databases as questions arise	18 (94.7)	26 (96.3)	0.66
- Read research articles in professional journals	14 (73.7)	22 (81.5)	0.39
- Read professional newsletters, list- serves, and research clippings	10 (52.6)	20 (74.1)	0.12
- Watch podcasts and webinars	5 (26.3)	13 (48.1)	0.12
- Attend continuing professional development programs	14 (73.7)	23 (85.2)	0.28
- Attend conferences and meetings of professional medical organizations	18 (94.7)	23 (85.2)	0.30
- Engage in clinical teaching	19 (100)	23 (85.2)	0.11
- Engage in research/scholarly activities	17 (89.5)	8 (29.6)	<0.001

M-mean; SD-standard deviation; na-not applicable

With respect to leisure-time exercise habits, more community-based physicians than academic physicians reported engaging in mild forms of exercising ≥4 times per week (37% vs. 31% of physicians) and moderate exercising at least once per week (82% vs. 68% of physicians). However, more academic physicians than community-based physicians reported engaging in strenuous forms of exercising at least once a week (58% vs. 47% of physicians). Only two physicians reported engaging in no leisure-time exercising. No significant difference in the leisure-time exercise scores of academic and community-based physicians was observed (academic: M (SD) = 15.74 (9.88); community-based: M (SD) = 14.89 (9.71); p = 0.77). Correlational analyses revealed that physicians’ leisure-time exercise scores were positively correlated with professional life satisfaction (r = 0.25; p = 0.058) and work engagement (r = 0.29; p = 0.028) and negatively correlated with work exhaustion (r = −0.34; p = 0.01).

## Discussion

This study provides an insight of lifelong learning activities and leisure-time exercise habits of physicians practicing in academic and community-based settings in Canada. Specifically, we have observed that the majority of physicians in this study engage in lifelong learning activities that offer in-person interactions with their colleagues and medical trainees, making physicians a part of a professional community of practice. We have also observed that leisure-time exercising of physicians was positively correlated with their professional satisfaction and work engagement and was negatively correlated with work exhaustion. Each of these findings is elaborated below.

To remain current in their practices, the majority of academic and community-based physicians reported that they exchange ideas/ask colleagues, engage in clinical teaching, and attend conferences and meetings of professional organizations. Historically, physicians have been a part of a connected professional community ( Frey, 2018). The structural changes, including the ongoing fragmentation of medicine and the growing size of practices, “have led to an increasing sense of professional loneliness that not only threatens the quality of clinical care by replacing personal discussions about patients but also poses risks to physician personal and professional wellbeing” ( Frey, 2018, p. 461). Our recent research also indicates that physicians’ need for relatedness in the workplace appears to be satisfied to a lesser extent ( Babenko, 2018). While there is a value of obligatory continuing education systems in ensuring the minimum requirements of knowledge and skills of practicing health care professionals, in-person interactions with colleagues and engaging in clinical teaching are valuable sources of lifelong learning ( Ding *et al*., 2018). Published literature indicates that clinical teaching has a positive impact on medical practice helping health care professionals stay current with medical knowledge, techniques, and guidelines ( Budden, Svechnikova and White, 2017), offering intellectual stimulation ( Ingham *et al*., 2015; Steinert and Macdonald, 2015), and enhancing morale and clinical practice ( Lochner, Wieser and Mischo-Kelling, 2012). With respect to in-person interactions with colleagues, Frey points out that “not valuing time with other physicians or making informal conversations possible leads to a soulless efficiency and professional isolation that drains physicians of our ability to help ourselves, help each other and help patients” ( Frey, 2018, p. 463). He calls for more social interaction during medical training and more emphasis on being part of a community of professionals.

In regard to leisure-time exercise habits, we have observed that community-based physicians in this study tended to engage in mild and/or moderate forms of exercising, whereas academic physicians engaged in strenuous forms of exercising in a typical week. In addition to clinical tasks, academic positions hold unique challenges, including research-related stress and competing demands. We speculate that engaging in exercise at higher levels of intensity may help academic physicians cope with stressors that are unique to their positions. It is also reassuring that almost all the physicians in this study engaged in leisure-time exercise several times a week. Furthermore, irrespective of the practice type, physicians who engaged in leisure-time exercise more often and/or at a higher intensity, experienced better professional wellbeing - that is, they were more professionally satisfied, more engaged and less exhausted at work. Published research indicates that health care providers, who do not engage in healthy lifestyles, including regular exercise, may not be counselling patients on the topics of exercise and healthy lifestyles ( Gnanendran *et al*., 2011; Klein, Guenther and Ross, 2016). For example, a recent Canadian study has revealed that there is a relationship between the personal health practices of physicians and their patient counseling practices, in terms of both frequency and topics of counseling ( Klein, Guenther and Ross, 2016). From this perspective, if CPD systems are explicitly designed to encourage health care providers to engage in healthy lifestyles, it will benefit not only their own wellbeing but also will enhance their patient counselling practices in preventative medicine. The importance of this cannot be overemphasized as benefits are expected for health care providers, their patients, and health systems.

## Conclusion

Designing CPD systems that: offer physicians in-person interactions with their colleagues, encourage physicians to engage in clinical teaching and support them in their teaching roles, and motivate physicians to adopt and maintain healthy lifestyles throughout their careers will have impact at various levels - the physicians as individuals, their patient populations, and the health systems in which they practice.

## Take Home Messages

• Lifelong learning activities and leisure-time exercise habits are important indicators of physician professionalism.
• In-person interactions with colleagues and involvement in clinical teaching are favoured by physicians as sources of lifelong learning.
• Participation in leisure-time exercise enhances physicians’ wellbeing, including professional satisfaction and work engagement.
• As such, these activities and habits should be encouraged, supported, and promoted within institutional culture and health systems in general.

## Notes On Contributors

Oksana Babenko, PhD, is an assistant professor and medical education researcher at the Department of Family Medicine, University of Alberta, Canada. Dr. Babenko collected the data, performed data analyses, wrote the first draft, and edited the manuscript. ORCID: https://orcid.org/0000-0003-2140-1551

Mao Ding, MA, is an undergraduate student at the Department of Chemistry, University of Alberta, Canada. At the time of this research, Ms. Ding worked as a summer research student under the supervision of Dr. Oksana Babenko at the Department of Family Medicine, University of Alberta. Ms. Ding contributed to data collection, including survey development and data management, interpretation of the study findings, and preparation of the manuscript. ORCID: https://orcid.org/0000-0002-0408-4278

Sudha Koppula, MD, MClSc, CCFP, FCFP, is an associate professor at the Department of Family Medicine, University of Alberta, Canada. Dr. Koppula contributed to the interpretation of the study findings and writing of the manuscript. ORCID: https://orcid.org/0000-0003-0744-4993
