# Supplementary material for: Lifelong learning practices and leisure-time exercise habits of academic and community-based physicians
Source: MedEdPublish (2016). 2019 Jan 29;8:21. [Version 1] doi: 10.15694/mep.2019.000021.1 (PMC10712484; doi:10.15694/mep.2019.000021.1)
Supplement: Peer review history

# Reviewer report: Reviewer response for version 1

**DOI:** 10.21956/mep.19762.r29670

**Published:** 2019-06-24

**Authors:** Ken Masters

**Affiliations:** 1 Sultan Qaboos University

This review has been migrated. The reviewer awarded 4 stars out of 5 An interesting paper on lifelong learning practices and leisure-time exercise habits of academic and community-based physicians. The paper gives a good background to the two areas under study, and the researchers have used several tools to gather data on the topic from Canadian doctors. Overall, the paper has been well-written, and the results clearly presented.Three small issues that need addressing:• The sample is relatively small, and this is a limitation that needs to be acknowledged.• The conclusion is extremely short, and undersells the paper. I would like to see this expanded somewhat to more appropriately reflect the paper's findings.• Given that many contextual aspects may influence the findings, and that the study was set in one country, it would probably be advisable to add “in Canada” to the end of the title.Nevertheless, and interesting and useful paper.

Reviewer Expertise:

NA

No decision status is available

# Reviewer report: Reviewer response for version 1

**DOI:** 10.21956/mep.19762.r29669

**Published:** 2019-02-04

**Authors:** Nandalal Gunaratne

**Affiliations:** 1 Faculty of Medicine, Wayamba University of Sri Lanka

This review has been migrated. The reviewer awarded 4 stars out of 5 Thank you for a well written, analysed and objectively concluded paper. While CPD activity and life long learning has been looked at by other authors, to connect it with their exercise is what makes this interesting.In addition to promoting life long learning in medical students and physicians, promoting healthy activities as a equally important adjunct can be supported by the findings in this paper.

Reviewer Expertise:

NA

No decision status is available

# Reviewer report: Reviewer response for version 1

**DOI:** 10.21956/mep.19762.r29668

**Published:** 2019-02-01

**Authors:** Helena Filipe

**Affiliations:** 1 Hospital of the Armed Forces/PL-EMGFA

This review has been migrated. The reviewer awarded 4 stars out of 5 I read this paper with interest and thank the Authors for their interest in publishing and thus collaborating in this special MedEdPublish themed on CPD.The manuscript is an original research focused on a relevant and applicable topic: physicians’ life long learning and wellbeing.The abstract is concise and summarizes the content that follows.Well written and supported, the introduction substantiates the authors’ research.The methods are clearly described and could be replicated.The Authors discuss thoroughly their experience.The positive relationship of inter-peer exchange of clinical practice experience with the physician wellbeing is good evidence to support the need of allocating time for peer to peer learning interaction while practicing as an effective CPD activity.The need of physicians to protect their well being as a professional duty, to develop an enjoyable clinical practice and find job satisfaction and to provide a good example augments their patients’ outcomes. This research highlights the relevant role of the organizational leadership to promote good clinical practices and quality improvement by facilitating work place learning environments with gains for the health care providers, the patients, the organization’s themselves and ultimately the health systems.The absence of difference between community based and academic professionals in their exercise pursuit to maintain their well being as we might suspect there would be, is an interesting finding. The conclusion is concise and drawn from the results.The take home messages are expertly taken out from the text.The table is well framed and informative.The manuscript overall reads well, focus on a relevant topic that healthcare professionals have increasingly to deal with, underlines a role and responsibility of the organizations to promote CPD in order to facilitate experiential learning environments and shows how the HCP well being can tie with patients outcomes. I compliment the Authors on their topic choice as for the well-conducted and described experience.

Reviewer Expertise:

NA

No decision status is available
